# First Report of a Histological Technique for Observing the Anatomy of the Epidermis of *Melinis minutiflora*

**DOI:** 10.1002/jemt.70010

**Published:** 2025-06-20

**Authors:** Josiane Costa Maciel, Cássia Michelle Cabral, Joice Mariana Santos Silva, Caique Menezes de Abreu, Fernanda Santos Oliveira, Iasmim Marcella Souza, Dayana Maria Teodoro Francino, Evander Alves Ferreira, Ricardo Siqueira da Silva, José Barbosa dos Santos

**Affiliations:** ^1^ Departamento de Agronomia Universidade Federal dos Vales do Jequitinhonha e Mucuri Diamantina Brazil; ^2^ Departamento de Ciências Biológicas Universidade Federal dos Vales do Jequitinhonha e Mucuri Diamantina Brazil; ^3^ Instituto de Ciências Agrárias Universidade Federal de Minas Gerais Montes Claros Brazil

**Keywords:** African grass, dissociation of leaf epidermis, histological technique, invasive species, leaf anatomy

## Abstract

This article describes precise and unpublished data on leaf anatomy of *Melinis minutiflora* P. Beauv. This species is an aggressive invasive plant that invades Environmental Protection Areas. Studies on the dissociation of the leaf epidermis using histological techniques could help to identify specific leaf characteristics that can significantly influence the success of invasion into new environments. This study identifies, for the first time, a histological technique that makes it possible to dissociate the entire leaf epidermis of the invasive species *M. minutiflora* in order to evaluate the anatomical structures in frontal view. The freehand paradermal cut, the hydrochloric acid and sodium hypochlorite methods and the epidermal impression technique were evaluated. The median region of each leaf was sectioned into samples of approximately 1 cm^2^ to dissociate the epidermis. The results demonstrate that the method with sodium hypochlorite was the one that best enabled the dissociation of the *M. minutiflora* epidermis in its entirety and the obtaining of semi‐permanent slides. This method is easy to perform, effective and ensures the preservation of the structural integrity of the cells, allowing a clearer and more detailed visualization of the cells and tissues under the microscope. This method also has the potential to be applied to plants that have stomata arranged in longitudinal rows and have morphological characteristics that make it difficult to observe the stomata. The histological technique of printing the epidermis with instant adhesive, although it has presented some negative points, such as distortion and deformation of the cells, is a useful and low‐cost technique for measuring characters of interest. These findings provide a better understanding of leaf anatomy and contribute to the accurate identification of invasive plant species and the development of effective control and management strategies.

## Graphical abstract

A paired comparison revealed that the sodium hypochlorite method offers several advantages over other techniques, better preserving anatomical structures, which facilitates detailed analysis.

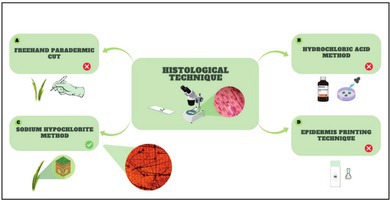

**Summary**

- The epidermis of *Melinis minutiflora* was dissociated using histological techniques.
- The hydrochloric acid technique did not allow the epidermis to be dissociated.
- The use of sodium hypochlorite is the most effective method for dissociating the epidermis of *Melinis minutiflora* leaves.

## 1 Introduction

*Melinis minutiflora* is a perennial grass, C4, native to Africa, has a high growth rate, is difficult to eradicate, and has few cultivars in Brazil (Barger et al. 2003; Oliveira and Reis 2024). It is considered an invasive species in South America, Central America, North America, Oceania, and Asia (GBIF 2024). The ecology of *M. minutiflora* is marked by its ability to adapt to different environmental conditions (Sena‐Souza et al. 2023) and its ability to invade different ecosystems, causing loss of biodiversity (Nogueira et al. 2019). Although the first report in Brazil dates back to 1812 (Parsons 1972), the anatomical aspects of these groups of non‐agricultural plants are still little explored.

The frequent invasions of *M. minutiflora* in open vegetation areas have become the main cause of threat to biological diversity, mainly in the Cerrado biome (neotropical savanna) (Damasceno and Fidelis 2020). In the Brazilian Cerrado, this invasive species dominates protected areas, such as the Brasília National Park (Zanin 2009), Emas National Park (França et al. 2007), Itirapina Ecological Station (Damasceno et al. 2018), Serra do Rola Moça State Park (Ribeiro et al. 2017), Biribiri State Park (Rocha et al. 2017), Pico do Itambé State Park, among others. Competitive mechanisms, such as rapid growth, biomass accumulation, high seed production, and tolerance to environmental climatic conditions, confer a competitive advantage compared to native species (Lannes et al. 2012).

Understanding the anatomy of invasive plants allows us to inform fundamental aspects about their mechanisms of tolerance and adaptation to environments (Alencar et al. 2020; Ribeiro et al. 2020) and their competitive power, in relation to native species (Santos et al. 2018; Chubar and Burundukova 2023). The use of anatomy in studies of invasive plants offers many advantages, such as the identification of characteristics that are associated with rapid plant growth, anatomical adaptations that confirm tolerance to disturbances, study of the morphology of organs such as leaves and roots, detection of cellular and tissue modifications, which serve as a subsidy for the investigation of invasion mechanisms (Krähmer et al. 2021; Manzoor et al. 2023). This is fundamental to understanding their growth, reproduction, and survival strategies (Ciccarelli and Bona 2022).

The methodological approach to dissociation of leaf epidermis through histological techniques must be a careful and precise process. The epidermis, as the outer layer of the leaf, can be carefully dissociated by some methods. There are histological techniques such as the use of hydrochloric acid, the Jeffrey method (nitric and chromic acid solution), the use of weak acids such as citric acid, freehand paradermal cutting, the potassium hydroxide technique, the epidermal impression technique, and the sodium hypochlorite method that can be used for this purpose (Johansen 1940; Segatto et al. 2004; Joffily and Vieira 2010; Gobbi et al. 2011; Mar‐Jiménez and Simón 2022).

When choosing a technique for separating the epidermis from grass leaves, the specific anatomy of the plant and the sensitivity of the leaves must be taken into account, adapting the procedures as necessary (Mauri et al. 2018). The dissociation of the epidermis of grasses is due to a complexity in the cellular structure of the mesophyll and epidermis cells (Sanchês et al. 2021). *Melinis minutiflora* has epidermal cells of different types and functions with bulliform cells distributed in bands, trichomes interspersed with stomata also distributed in bands (Bohley et al. 2019). In the mesoderm parenchymal cells, forming the chlorophyll parenchyma with mesophyll cells, bundle sheath cells, and vascular bundles presenting many fibers (Baird et al. 2024), making it difficult to isolate the epidermis without damaging paradermal cells or structures. Therefore, finding the correct and precise technique ensures the preservation of the integrity of the epidermal cells for later analyses.

Current work specifically involving the leaf anatomy of *M. minutiflora* , with regard to the characteristics of the epidermis, such as shape, number of stomata, and trichomes, is scarce in the literature. Considering the relevance of these characteristics for the ecology and invasive success of the species, there is a gap to be explored. Therefore, the identification of an appropriate histological technique may help identify potential correlations between the anatomy of the epidermis and invasive success, and thus adjust more effective and sustainable control strategies against this invasive plant.

The objective of this work was to identify a histological technique that allows the dissociation of the leaf epidermis of the invasive species *M. minutiflora* in its entirety for evaluation of the anatomical structures in frontal view.

## 2 Material and Methods

### 2.1 Plant Material

**FIGURE 1:**
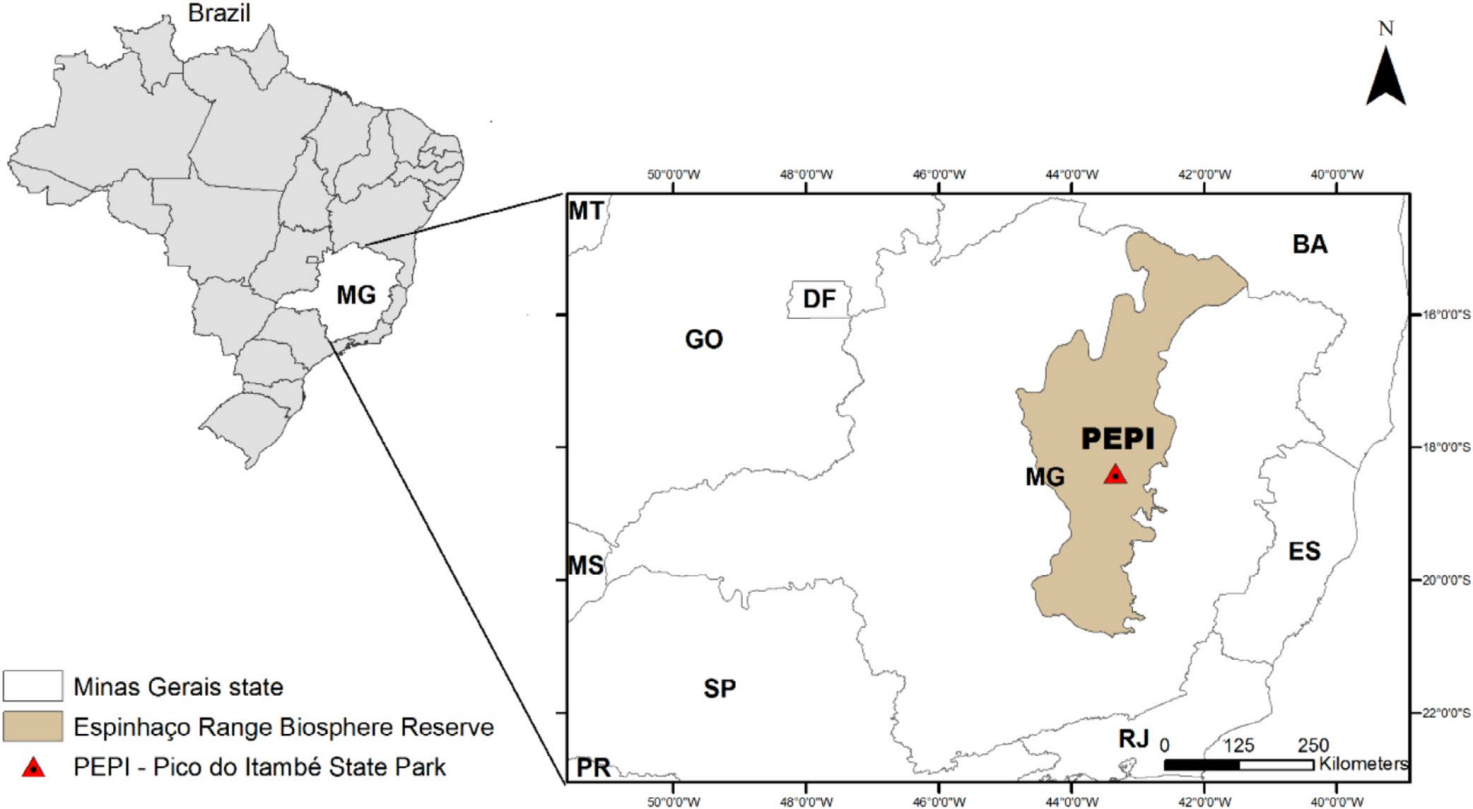
Environmental conservation unit, Pico do Itambé State Park in the central portion of the Serra do Espinhaço in the state of Minas Gerais (MG), Brazil.

Leaf samples of *M. minutiflora* were collected at Pico do Itambé State Park—PEPI (18°24′00.1″S and 43°19′22.3″W; 1573 m) in 2022. The park is located in the upper region of the Alto Vale do Jequitinhonha, in the central portion of the Serra do Espinhaço, Minas Gerais (MG), Brazil (Figure 1). The PEPI covers part of the municipalities of Serro, Serra Azul de Minas, and Santo Antônio do Itambé, in the state of Minas Gerais, Brazil, and is a quartzite massif, representing one of the highest points of the Serra do Espinhaço. The region's climate regime is typically tropical, with temperatures between 18°C and 19°C and an average annual occurrence ranging from 1.250 to 1.550 mm (Cardoso et al. 2020).

The leaves were collected in the morning. Three tillers were selected from each plant, totaling five plants. Leaves that were fully expanded and had a visible ligule were considered for collection, totaling 15 samples.

The leaves were collected between the third and fourth node, counting from the apex to the base. *Melinis minutiflora* leaf tissues were fixed with FAA 70 GL (Gay‐Lussac) (5% formaldehyde to 40%; 5% glacial acetic acid and 90% ethyl alcohol to 70%) for 48 h, at an approximate ratio of 20 times the volume of fixative/volume of plant tissue. The analyses were performed in the Plant Anatomy laboratory of the Department of Biological Sciences of the Universidade Federal dos Vales do Jequitinhonha e Mucuri—UFVJM in Diamantina, Minas Gerais, Brazil. The samples were removed from the FAA fixative solution and kept in 70 GL ethanol solution (Gay‐Lussac) (Johansen 1940), according to techniques for making semi‐permanent slides in plant anatomy. The median region of each leaf was sectioned into a sample of approximately 1 cm^2^, using a stainless steel blade (11 × 9 × 1.1 cm), and a Petri dish (100 × 20 cm) as a support.

### 2.2 Anatomical Procedures

Four histological techniques were tested for making semi‐permanent slides: (1) freehand paradermal cut; (2) hydrochloric acid (HCl) method; (3) sodium hypochlorite (NaClO) method; (4) epidermal impression technique. The choice of techniques took into account the resources available in the laboratory.

### 2.3 Freehand Paradermal Cutting

**FIGURE 2:**
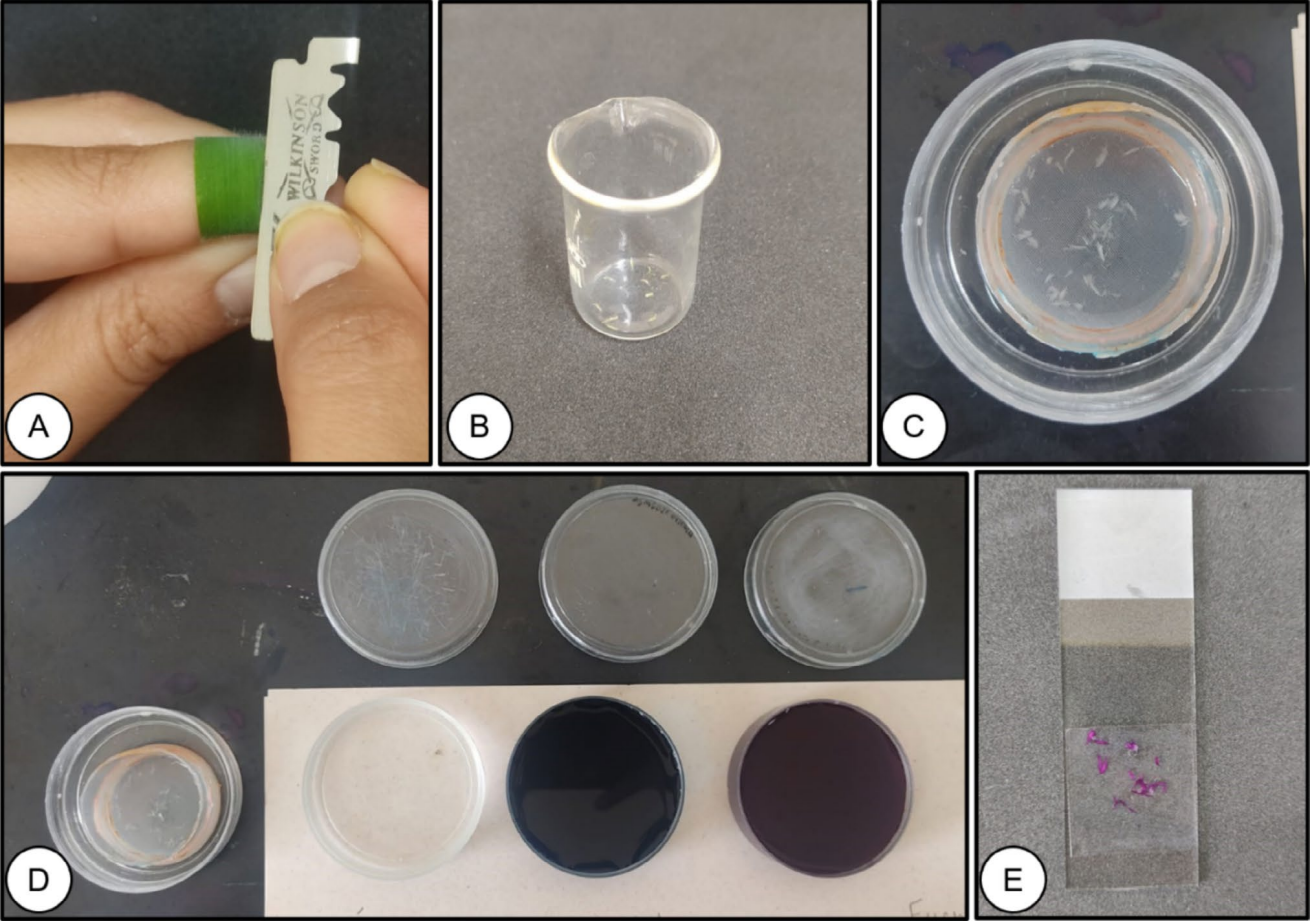
Obtaining freehand paradermal sections in the adaxial and abaxial parts of the leaf blade of *Melinis minutiflora* . Handling of the *M. minutiflora* leaf between the thumb and index finger (A), storage of the sectioned tissues (cuts) in a 10 mL glass beaker (B), storage of the tissues in a retention mesh for clarification (C), staining of the sectioned leaf tissues with Alcian Blue and Fuchsin (D), sealing of the tissues in a semi‐permanent histological slide (E).

The leaf was placed between the thumb and index finger to make the cut (Figure 2A). The leaf epidermis was removed with the aid of a stainless steel blade (Macedo 1997), sliding it gently and continuously over the leaf surface. The sections were placed in a glass beaker (Uniglas brand) with distilled water and, with the aid of a magnifying glass, those with translucent potential were selected (Figure 2B) and then, with the aid of a number zero brush, they were placed in a fine mesh sieve (Figure 2C). To dissociate the epidermis, the sections were immersed in a 50% sodium hypochlorite (NaClO) solution (active chlorine content between 2.0% and 2.5% w/w) for 20 min, then washed with distilled water (using a spray bottle) until there was no more odor. Subsequently, the sections were inserted into a 10% acetic acid (CH_3_COOH) solution for 1 min, with no need for the washing step.

The sections were stained with Alcian Blue for 5 min, followed by a triple wash with distilled water; Basic Fuchsin for 5 s and finished by a triple wash (Figure 2D).

A drop of 50% glycerin solution (v/v^−1^) was added to a glass slide and, using a number zero brush, the cuts were placed equidistantly. Glass coverslip (20 × 20 mm) was added to the tissues (sections) to seal them (Figure 2E).

### 2.4 Hydrochloric Acid Method

**FIGURE 3:**
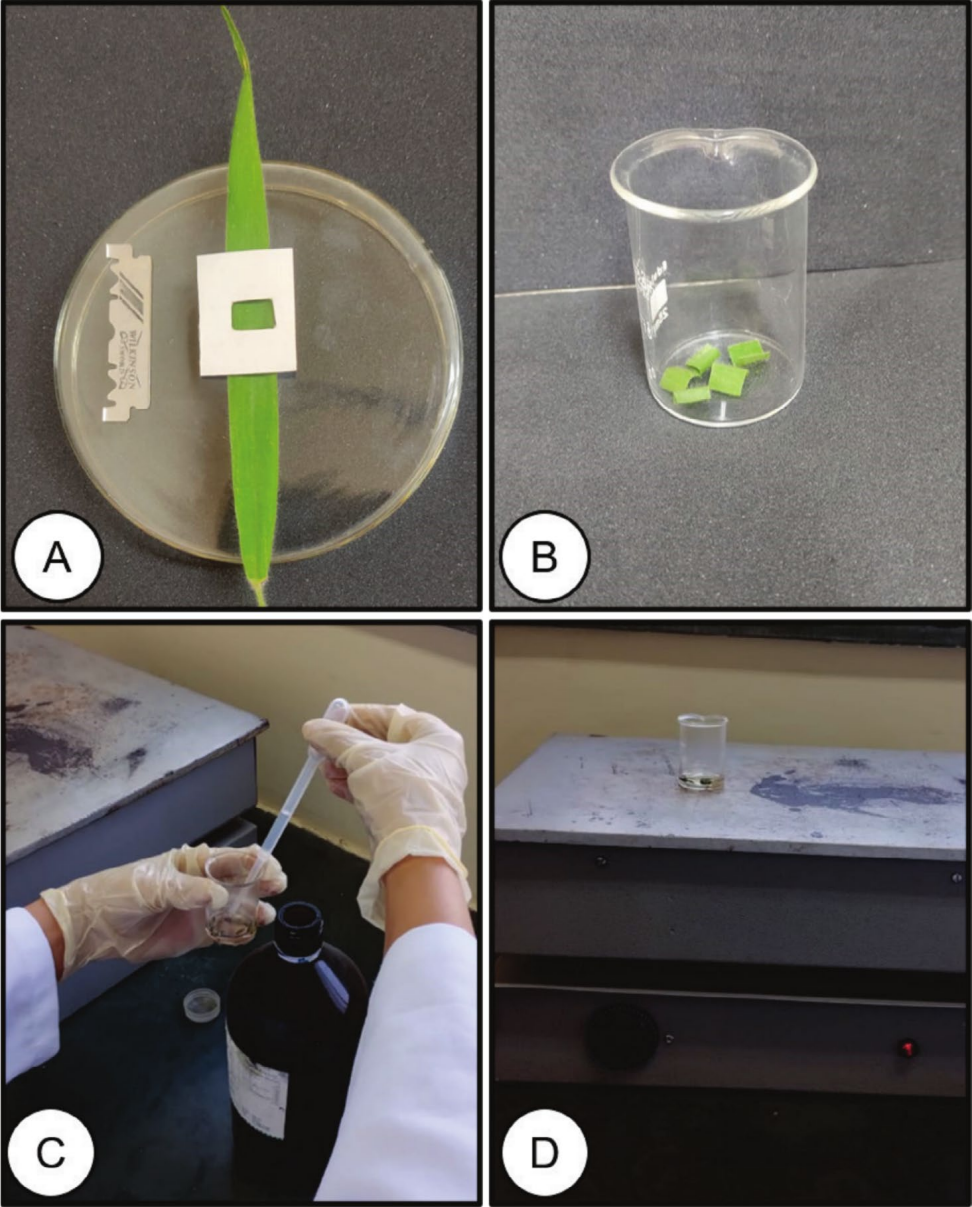
Procedure for sectioning leaf tissue of *Melinis minutiflora* using the hydrochloric acid method. Sectioning of subsamples (1 cm^2^) in the median portion of the leaf (A), immersion of the leaf tissue in a 50 mL glass beaker containing hydrochloric acid (B and C), heating, boiling and product of the leaf tissue (D).

Five samples composed of a 1 cm^2^ section of the median region of the leaf (Figure 3A) were isolated using a stainless steel blade. To dissociate the epidermis, the samples were placed in glass beakers containing 6 mL of HCl (Figure 3B,C) and boiled at 100°C using a hot plate (Figure 3D) to disintegrate the mesophyll and dissociate the epidermis for 20 min.

### 2.5 Sodium Hypochlorite Method

**FIGURE 4:**
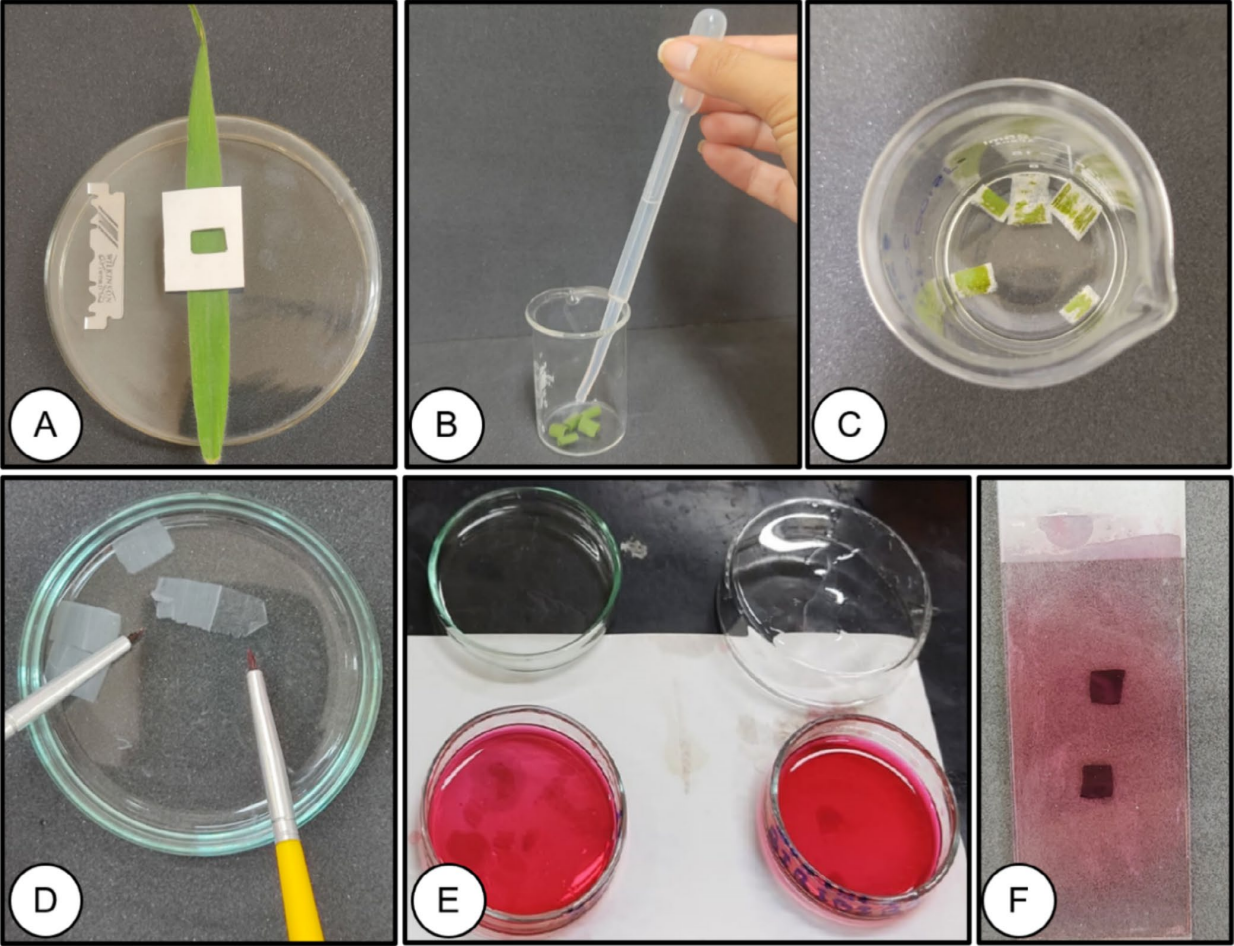
Procedure for sectioning leaf tissue of *Melinis minutiflora* using the sodium hypochlorite method. Sectioning of sub‐samples (1 cm^2^) in the median portion of the leaf (A), samples stored in a 50 mL glass beaker containing sodium hypochlorite (B and C), separation of leaf epidermis (D), staining of tissues with Safranin (E), sealing of tissues in a semi‐permanent histological slide (F).

Five samples composed of a 1 cm^2^ section of the median region of the leaf (Figure 4A) were isolated using a stainless steel blade. To dissociate the epidermis, the samples were placed in a glass beaker and 6 mL of sodium hypochlorite (the active chlorine content was between 2.0% and 2.5% w/w) were added for 24 h, followed by a triple wash with distilled water (Figure 4B,C).

The mesophyll separation was performed in a Petri dish, adding 4 mL of distilled water and using two number zero brushes (Figure 4D). Staining was performed with 20 drops of 1% Safranin solution in 50 GL alcohol for 24 h (Figure 4E). The fragments were mounted between a slide and coverslip for visualization, and 50% glycerin was used as the embedding medium (Figure 4F).

### 2.6 Epidermis Printing Technique

**FIGURE 5:**
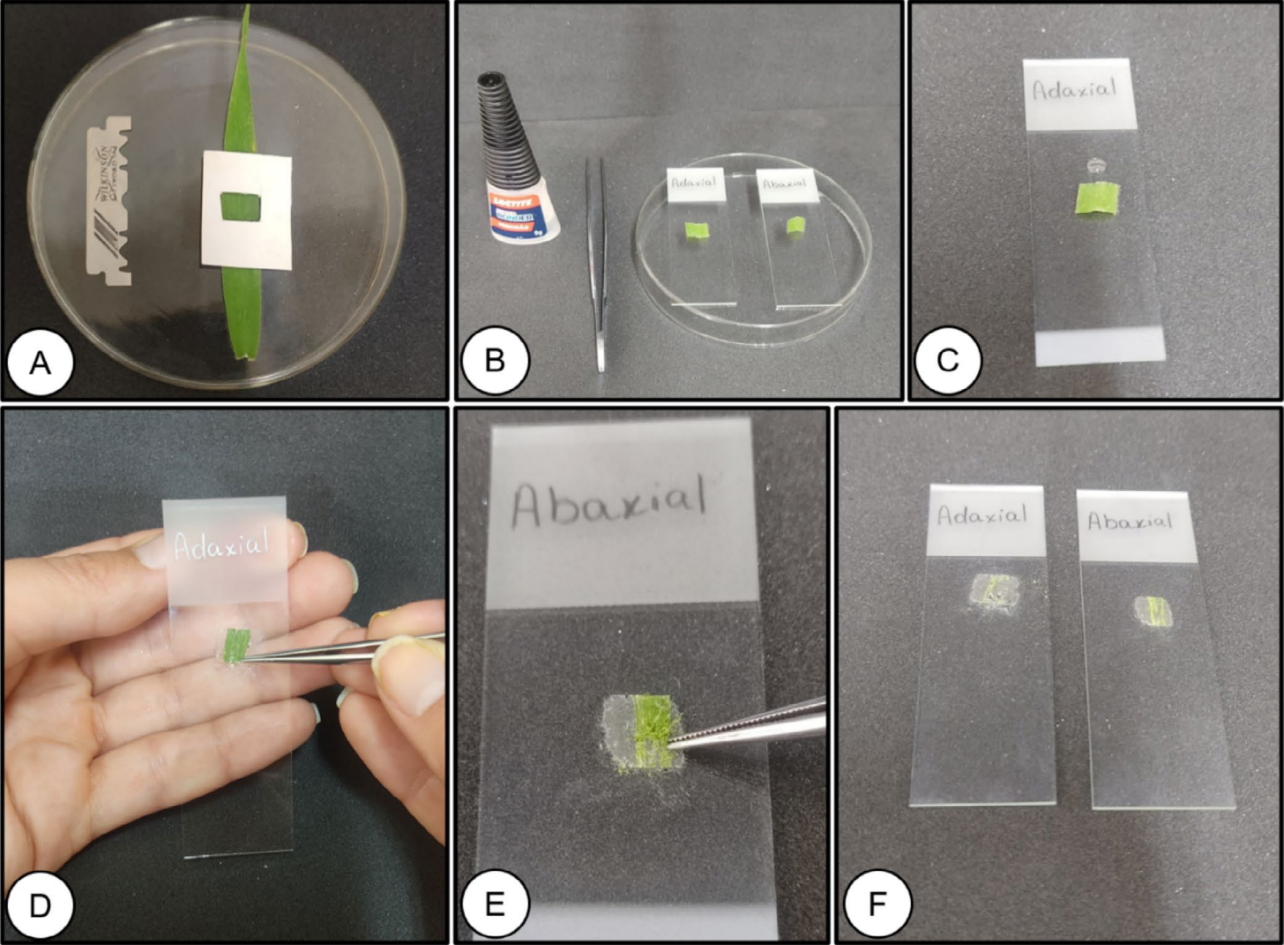
Procedure for sectioning leaf tissue of *Melinis minutiflora* using the epidermal impression method. Sectioning of subsamples (1 cm^2^) in the median portion of the leaf (A), arrangement of the abaxial and adaxial surfaces on the slides (B), preparation of the instant adhesive for printing the epidermis (C), printing of the epidermis in instant adhesive (D and E), result of the formation of histological slides by the epidermis printing method (F).

Two 1 cm^2^ samples from the median region of the leaf blade from both surfaces (adaxial and abaxial) were used (Figure 5A,B). A drop of universal instant adhesive (cyanoacrylate ester—Super Bonder) was added to a glass slide, and one of the samples was pressed for 3 min (previously tested time) (Figure 5C) to print the epidermal structures in the layer. The separation of the leaflet from the glass slide was performed using fine‐tipped histological forceps (Figure 5D,E). The use of a coverslip was not necessary for observation under an optical microscope (Figure 5F).

### 2.7 Photodocumentation

The semi‐permanent slides were observed under an optical microscope (Leica model DM500) and photomicrographed with the camera (Pocco X3 Pro) attached to the microscope.

## 3 Results

**FIGURE 6:**
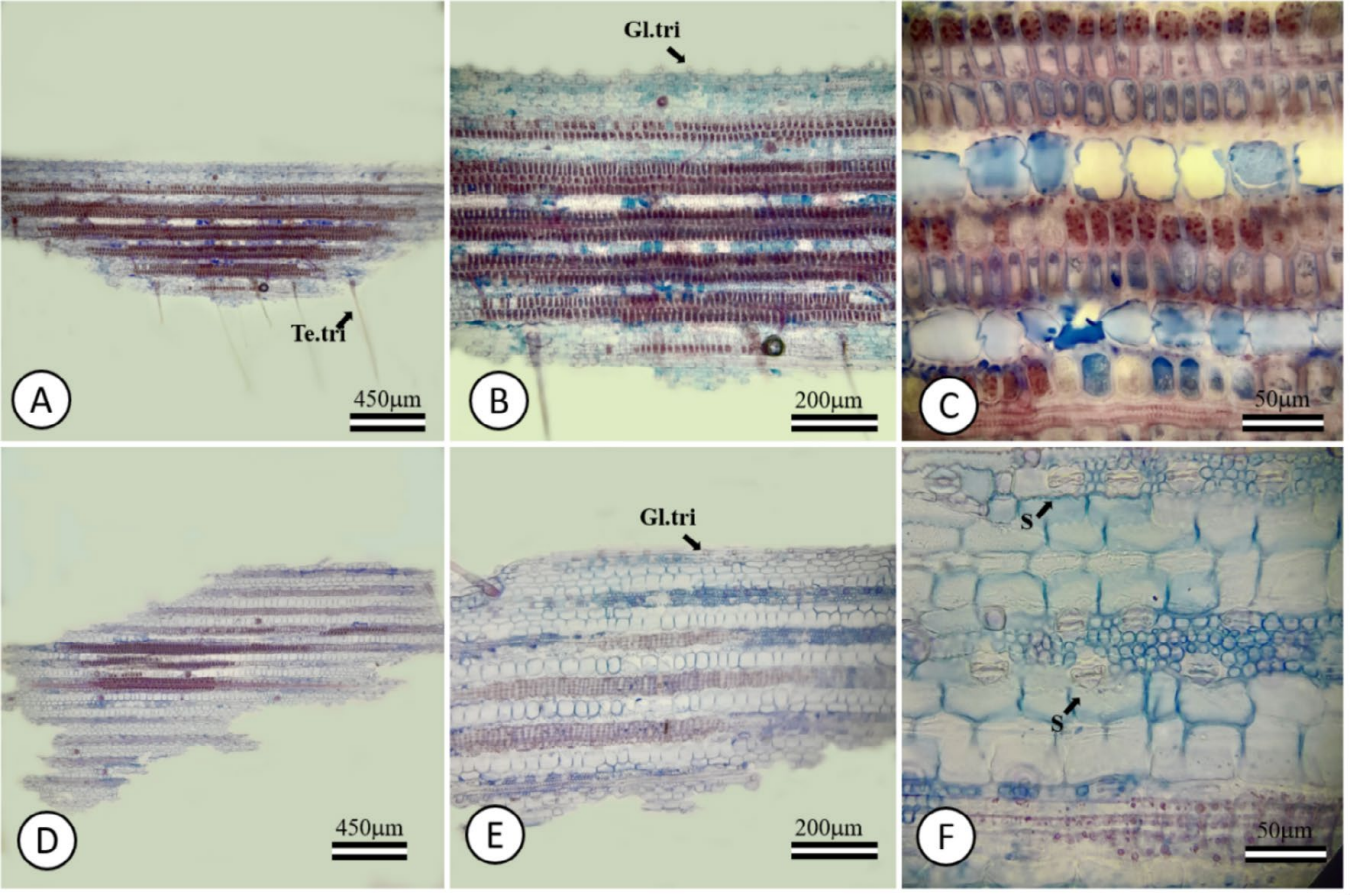
Photomicrograph of the paradermal section of the leaf blade of *Melinis minutiflora* . Adaxial epidermis showing the glandular and tector trichomes (A–C), abaxial epidermis showing glandular trichomes and stomata (D–F). Gl.tri, glandular trichome; S, stomata; Te.tri, tector trichomes.

The results of this study showed that, for the freehand histological cutting technique, the quality and precision of the cuts were not adequate. This technique resulted in sections of varying thicknesses, which made it difficult to accurately analyze cellular structures. In addition, it was not possible to remove the mesophyll, and this prevented microscopic analysis and interpretation of the results. Stomata, glandular trichomes, and tectors were some of the epidermal structures observed; however, it was not possible to calculate their density (Figure 6).

**FIGURE 7:**
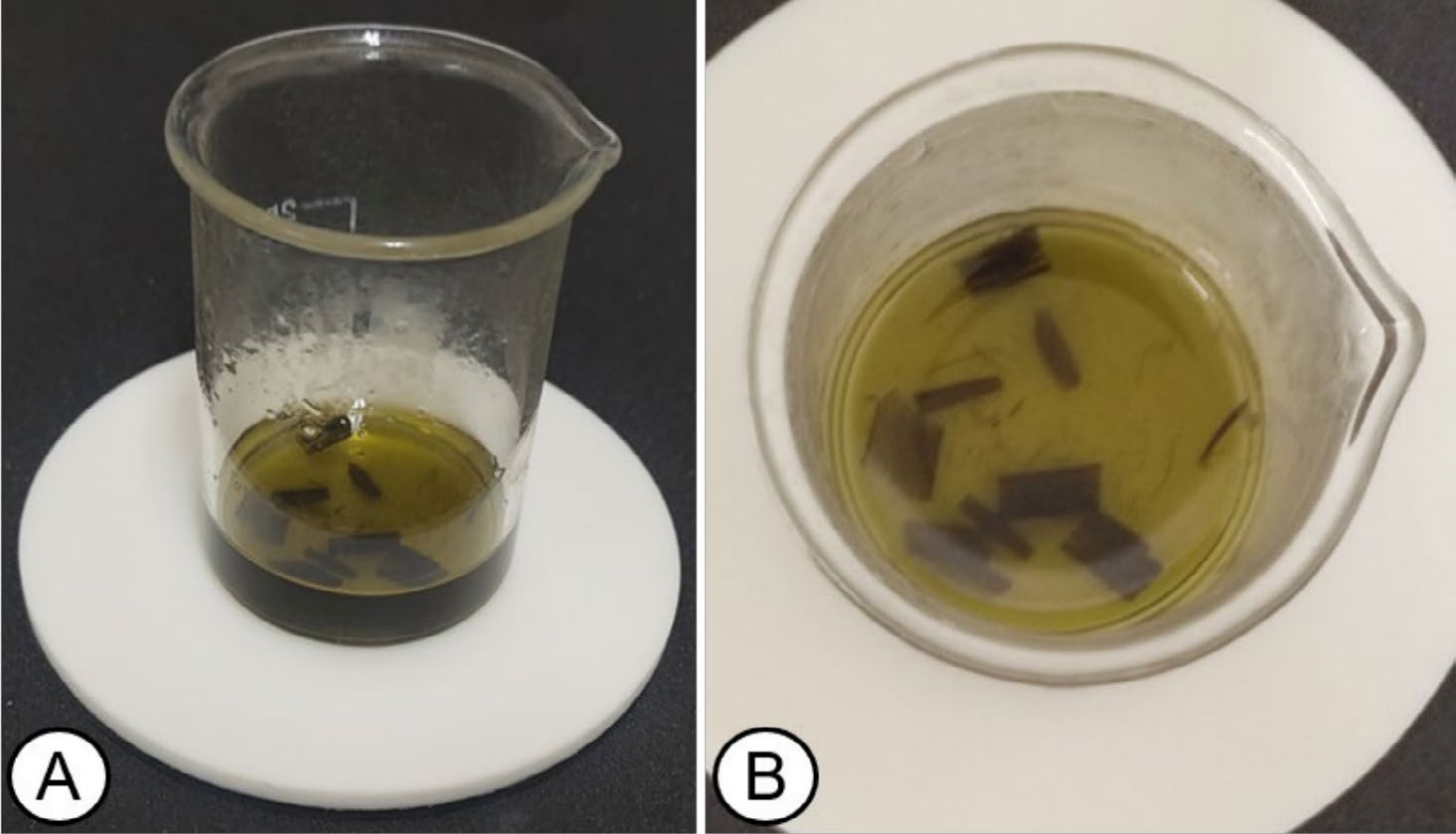
Destructuring of the cells and tissues of the leaf blade of *Melinis minutiflora* in a 50 mL glass beaker (A and B).

The histological technique that included immersion of the leaves in hydrochloric acid did not allow dissociation of the epidermis. Boiling the samples in hydrochloric acid for 20 min resulted in complete degradation of the tissues (Figure 7). This prevented the visualization of the epidermal structures and compromised the ability to analyze the samples. An adjustment in time could be an alternative for the suitability of this method.

**FIGURE 8:**
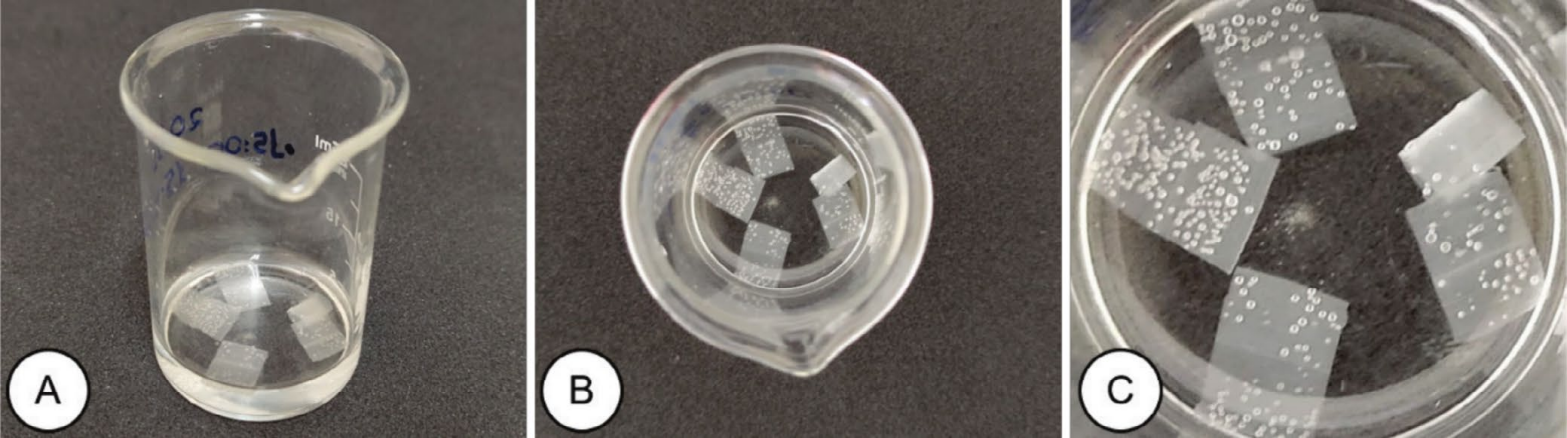
Tissue separation process. 1 cm^2^ segments of the median region of the leaves in a 50 mL glass Beaker containing sodium hypochlorite (active chlorine content between 2.0% and 2.5% w/w) to make the epidermis transparent, after 24 h (A and B). Higher magnification to emphasize air bubbles between the epidermis (C).

The method with sodium hypochlorite was the only one that allowed the dissociation of the *M. minutiflora* epidermis in its entirety and the obtaining of semi‐permanent slides. The reactions that occur in the mesophyll cells, in interaction with the sodium hypochlorite solution, result in the formation of air bubbles that contribute to the dissociation of the adaxial and abaxial epidermis (Figure 8).

**FIGURE 9:**
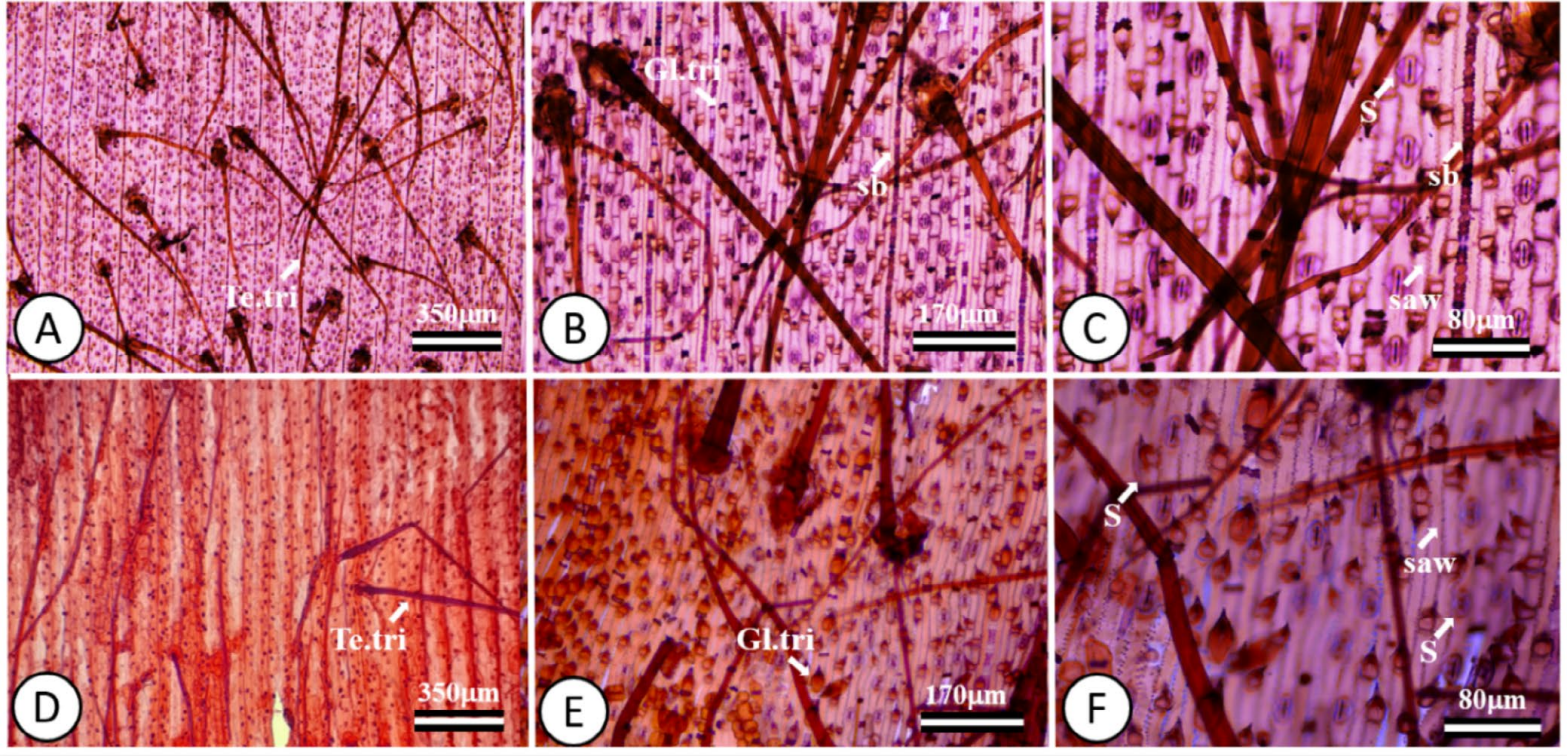
Photomicrograph of the paradermal section of the leaf blade of *Melinis minutiflora* . Adaxial epidermis (A–C), abaxial epidermis (D–F), details of tector and glandular trichomes (A, B, D, E), sinuous anticlinal wall and stomata (C, F), and siliceous bodies (B, C). Gl.tri, glandular trichome; S, stomata; saw, sinuous anticlinal wall; sb, siliceous bodies; Te.tri, tector trichomes.

The leaf anatomy of *M. minutiflora* has typical characteristics of the Poaceae family, with emphasis on the contour of the epidermal cells, trichomes, and stomata. The adaxial (Figure 9A–C) and abaxial (Figure 9D–F) faces of the epidermis exhibit glandular trichomes and tector trichomes (Figure 9A,B,D,E), sinuous anticlinal cell walls, which confer an irregular and adaptive structure (Figure 9C,F), stomata of epidermal guard cells in a bar‐like shape (Figure 9C,F) and siliceous bodies (Figure 9B,C). The common epidermal cells have a polygonal shape, which contributes to the strength and functionality of the leaf. In detail, it was possible to visualize the siliceous cells or siliceous bodies with a serrated rod shape, between the veins. There is the presence of large quantities of glandular microtrichomes in the costal zones, mainly on the abaxial surface, and elongated tector macrotrichomes attached to the veins. The leaves of *M. minutiflora* are amphistomatic, i.e., they have stomata on both sides, and quantification revealed that stomata density is significantly higher on the adaxial side.

This technique made it possible to count epidermal structures such as stomata and trichomes. Stomatal and trichome density, stomatal index, and stomatal functionality could be measured with this technique. The stomatal and trichome density calculated by the number of stomata per mm^2^ of leaf area varied between the adaxial and abaxial epidermis. The adaxial stomatal density was greater than the abaxial one, with an average adaxial stomatal density of 407.66 mm^2^ and an abaxial stomatal density of 290.25 mm^2^. As for the density of tector trichomes, the highest value was in the abaxial epidermis with an average of 19.25 mm^2^ when compared to the adaxial with 4.77 mm^2^. The stomatal index was calculated using the formula proposed by Cutter (1986): SI = NS/(EC + NS) × 100 where SI is the stomatal index, NS and EC are the number of stomata and the number of epidermal cells per unit area, respectively, in the microscopic field of view, with the values expressed as a percentage (%). The adaxial stomatal index of *M. minutiflora* was 55.90%, and the abaxial stomatal index was 25.22%. For stomatal functionality calculated by dividing the polar diameter by the equatorial diameter of the stomata, according to Castro et al. (2009), the average value was 4.98.

**FIGURE 10:**
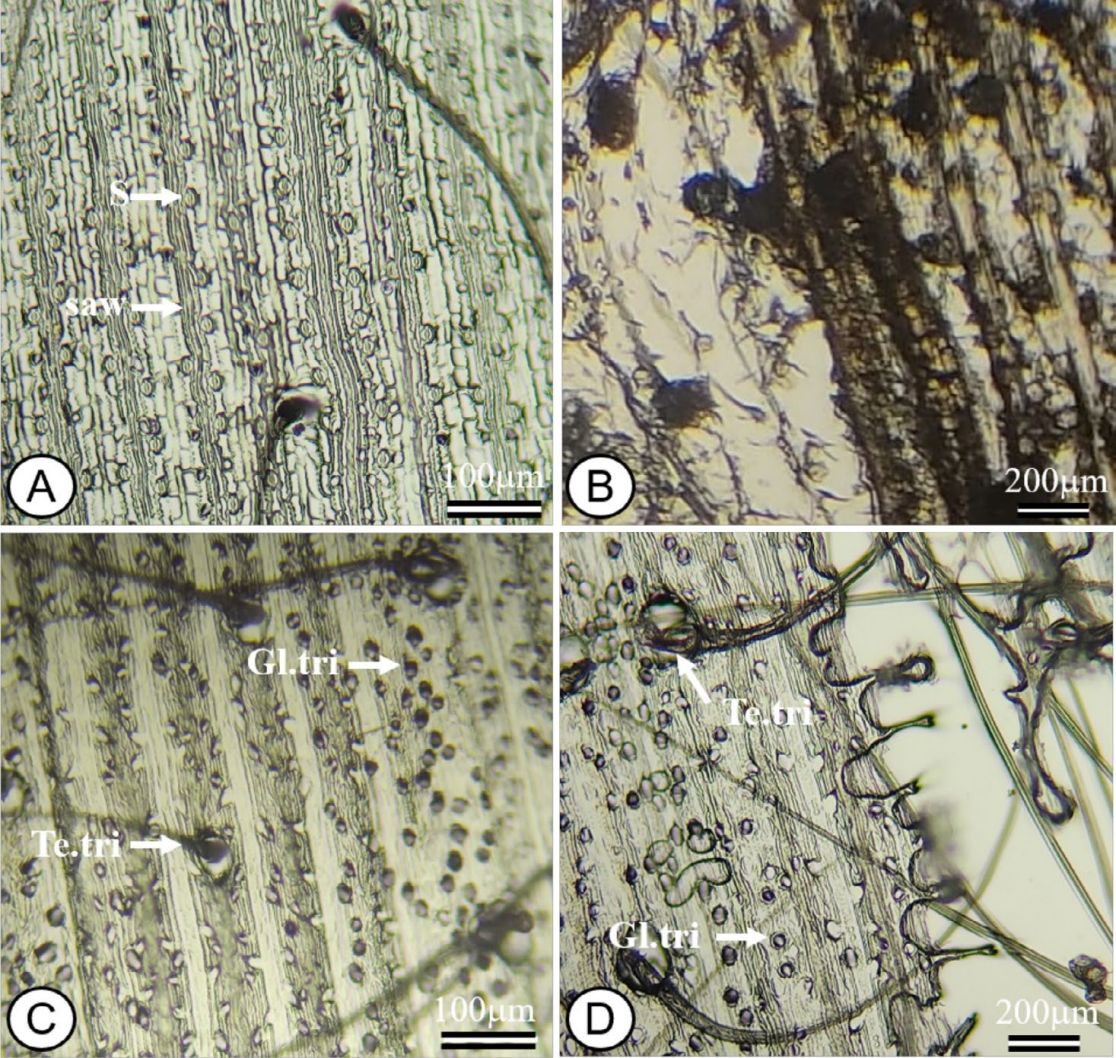
Photomicrograph of the paradermal section of the leaf blade of *Melinis minutiflora* . Adaxial epidermis (A, B) with detail of the stoma and sinuous anticlinal wall and abaxial epidermis (C, D) with detail of the glandular trichome and tector trichome. Adaxial (B) and abaxial (D) epidermis with flattening and cell deformation. Gl.tri, glandular trichome; S, stomata; saw, sinuous anticlinal wall; Te.tri = tector trichomes.

The technique of printing the epidermis with instant adhesive, although it allowed the visualization of some of the structures of the epidermis, such as the stomata, sinuous anticlinal wall (Figure 10A), tector trichome, and glandular trichome (Figure 10B), presented some negative points that impacted the quality of the results. During removal of the leaf surface, there was distortion of the cell morphology, flattening, and deformation of the cells (Figure 10C,D), which compromised the accuracy in observing the cell structures on some slides.

## 4 Discussion

Standardizing the thickness of the cut is essential in histological techniques to ensure consistent results, especially when the aim is to verify changes according to the characteristics of the environment, such as different altitudes, temperatures, humidity, and photoperiod (Joffily and Vieira 2010; Feng et al. 2022; Yang et al. 2022). There are reports that for qualitative analyses, where the aim is to describe and identify the structural characteristics of the leaves, such as cell type, tissue organization, presence of stomata and trichomes, small variations in the thickness of freehand sections are acceptable (Dickison 2000; Cutler et al. 2008), as long as the essential cell structures can be observed and described. With this technique, it is difficult to maintain standardization, which can lead to variations between different preparations (Lemos and Chaves 2022). In addition, the lack of full control over the parameters during freehand cutting can result in limited reproducibility between different experiments or between different executors (Lemos and Chaves 2022). Removing the mesophyll in manual cuts is also a challenging task due to the complexity of the leaf structure. In plants with a compact mesophyll, i.e., with few intercellular spaces, this is even more difficult. The mesophyll is the central part of the leaf, positioned between the upper and lower epidermis, and its thickness basically makes up the layers of palisade parenchyma and spongy parenchyma (Zekri et al. 2024). Their removal in this technique can result in variations between different attempts, making it difficult to obtain consistent results. Paradermal freehand cutting, despite its low cost, was unfeasible for carrying out anatomical studies of the leaf epidermis of *M. minutiflora* , although it has been used successfully in species from the Sterculiaceae (Nakayama et al. 1996), Arecaceae (Leite and Scatena 2001), and Pteridaceae (Graçano et al. 2001) families.

The degradation of tissues in the hydrochloric acid method can be explained by the strong action of this acid causing destruction of cellular structures and tissues (Valerio et al. 2013), resulting in the loss of morphological and anatomical information, and making it impossible to prepare slides. This method requires care to ensure that only the underlying cells are affected, leaving the epidermis intact. It is extremely important to carefully control the acid concentration and time period to avoid damage to the epidermis. The use of the hydrochloric acid technique may be valid when the objective is the total separation of cells (maceration technique) (Almeida et al. 2009; Sá et al. 2016).

The epidermis clarification and dissociation technique using sodium hypochlorite was effective in removing chlorophyll and other substances that hinder the visualization of cellular structures, as well as promoting the destructuring of the mesophyll (García‐Gutiérrez et al. 2020; Gobbi et al. 2011), for the complete dissociation of the leaf epidermis. This method preserved the morphology of cells and epidermal cellular structures such as glandular trichomes, tector trichomes, siliceous bodies and stomata, allowing a more precise analysis. It is important to highlight that this technique used for this species with the appropriate concentration of sodium hypochlorite and the period of time is unprecedented.

The density of stomata and trichomes is inversely related, that is, as stomatal density decreases, the number of trichomes increases (Batagin‐Piotto et al. 2012). This may be due to the functions of trichomes, which secrete substances capable of protecting plants against predators and can also act indirectly to save water, reflecting sunlight and, consequently, reducing the temperature of the leaves (Larcher 2000). This analysis was also reported in works focusing on the survival of plants of the Anacardiaceae family in environments with water limitations (Mercado et al. 2024), and on the identification of characters with taxonomic value and ecological importance of plants of the Convolvulaceae family (Dos Santos et al. 2024).

The organization of the epidermal structures in *Melinis minutiflora* (fat grass) shows characteristics typical of the Poaceae family. Some comparative epidermal structures are: Buliform cells, Stomata (the stomatal arrangement can vary) the type of stomata, trichomes. As well as the internal organization of the leaf, characterized as a homogeneous mesophyll with large, spaced cells. This arrangement of the mesophyll favors the entry and percolation of hypochlorite in samples immersed in the solution, benefiting the dissociation of the epidermis in an integrated and isolated way, generating samples with easier visualization.

The epidermal printing technique, although an interesting, fast and useful approach for collecting anatomical data in the field and without the requirement for fixation and staining processes (Gitz and Baker 2009; Batagin‐Piotto et al. 2012; Manzani Lisboa et al. 2024), can result in variations in the moment of removal of the leaf from the slide, causing potential damage to the epidermal structures. According to a study in another Poaceae ( *Paspalum notatum* ), visualization and counting of stomata is not an easy step in frontal view due to the morphology of the leaves, as they have a very sinuous leaf blade on the adaxial surface and the papillae or trichomes can cover the stomata (Santos et al. 2001), and in this case it was enough to make it difficult to visualize the stomata on the abaxial surface of *M. minutiflora* .

Preserving the integrity of anatomical structures is important, and this is relevant in invasion ecology studies that require high precision in the reproduction of impressions. Thus, this technique becomes less efficient in terms of reproducibility. However, it was an efficient technique in studies with *Solanum tuberosum* (Segatto et al. 2004).

## 5 Conclusion

The method with sodium hypochlorite enabled the dissociation of the *M. minutiflora* epidermis and ensured the preservation of the structural integrity of the cells. The recognition of the type, number and individual size of cells and the evaluation of the anatomical structures of *M. minutiflora* was possible with this method. In addition to allowing correct visualization and analysis, it has the advantage of dissociating the epidermis in a shorter time, especially if the number of samples is high. This method has the potential to be applied to plants that have stomata arranged in longitudinal rows and have morphological characteristics that make it difficult to observe the stomata. This method can be applied to other types of grass that are also invasive in protected areas, such as *Urochloa decumbens* (Stapf) R. D. Webster and *Panicum maximum* Jacq. It can also be used when the objective is to analyze taxonomy, plant physiology, studies of plant adaptation to different environments and different cultivation conditions such as temperature, light and water stress. The histological technique of printing the epidermis with instant adhesive, although it has presented some negative points, such as distortion and deformation of the cells, is a low‐cost technique for visualizing characters of interest. Our study generates relevant insights into the use of accurate histological techniques for the detailed study of the leaf anatomy of *M. minutiflora* .

## Author Contributions

**Josiane Costa Maciel:** conceptualization, formal analysis, investigation, methodology, validation, writing – original draft, writing – review and editing. **Cássia Michelle Cabral:** formal analysis, investigation, methodology, validation, supervision, writing – original draft, writing – review and editing. **Joice Mariana Santos Silva:** investigation, methodology. **Caique Menezes de Abreu:** investigation, writing – original draft, writing – review and editing. **Fernanda Santos Oliveira:** methodology, software. **Iasmim Marcella Souza:** software, writing – original draft. **Dayana Maria Teodoro Francino:** formal analysis, methodology, resources, supervision, writing – original draft, writing – review and editing. **Evander Alves Ferreira:** writing – original draft, writing – review and editing. **Ricardo Siqueira da Silva:** supervision, writing – original draft, writing – review and editing. **José Barbosa dos Santos:** methodology, resources, supervision, writing – original draft, writing – review and editing.

## Conflicts of Interest

The authors declare no conflicts of interest.

## Data Availability

The data that support the findings of this study are available from the corresponding author upon reasonable request.
